# Genome wide analysis of common and specific stress responses in adult drosophila melanogaster

**DOI:** 10.1186/1471-2164-5-74

**Published:** 2004-09-30

**Authors:** Fabrice Girardot, Véronique Monnier, Hervé Tricoire

**Affiliations:** 1 Institut Jacques Monod, 2 place Jussieu, 75251 Paris, France; 2 Present address: Equipe de Biologie Virtuelle, UMR 6543, Universilé de Nice, Parc Valrose, 06108 Nice Cedex 2, France

**Keywords:** *Drosophila*, microarrays, proteasome, P450, GT, stress.

## Abstract

**Background:**

During their life, multicellular organisms are challenged with oxidative stress. It is generated by several reactive oxygen species (ROS), may limit lifespan and has been related to several human diseases. ROS can generate a wide variety of defects in many cellular components and thus the response of the organism challenged with oxidative stress may share some features with other stress responses. Conversely, in spite of recent progress, a complete functional analysis of the transcriptional responses to different oxidative stresses in model organisms is still missing. In addition, the functional significance of observed transcriptional changes is still elusive.

**Results:**

We used oligonucleotide microarrays to address the specificities of transcriptional responses of adult Drosophila to different stresses induced by paraquat and H_2_O_2_, two oxidative stressors, and by tunicamycin which induces an endoplasmic reticulum (ER) stress. Both specific and common responses to the three stressors were observed and whole genome functional analysis identified several important classes of stress responsive genes. Within some functional classes, we observed that isozymes do not all behave similarly, which may reflect unsuspected functional specificities. Moreover, genetic experiments performed on a subset of lines bearing mutations in genes identified in microarray experiments showed that a significant number of these mutations may affect resistance of adult Drosophila to oxidative stress.

**Conclusions:**

A long term common stress response to paraquat- or H_2_O_2_-induced oxidative stresses and ER stress is observed for a significant number of genes. Besides this common response, the unexpected complexity of the stress responses to oxidative and ER stresses in Drosophila, suggest significant specificities in protective properties between genes associated to the same functional classes. According to our functional analysis, a large part of the genome may play a role in protective mechanisms against oxidative stress in Drosophila.

## Background

Cells are frequently submitted to exogenous or endogenous stresses. In aerobic cells, reactive oxygen species (ROS), produced by respiration and other biological processes, are a major source of endogenous stress. These ROS include the superoxide radical (O_2_•^-^), hydrogen peroxide (H_2_O_2_) and the highly reactive hydroxyl radical (OH•). Increased endogenous production, exposition to exogenous sources of ROS or reduction in antioxidant defense capacity cause molecular damages such as alterations in proteins, lipids and DNA, and may lead to cell death. Oxidative stress is believed to limit the lifespan of multicellular organisms [1,2] and oxidative lesions have been implicated in several human cardiovascular and neurodegenerative diseases [3]. A better understanding of the *in vivo *responses to oxidative stress is thus of major fundamental and practical importance.

Much data describing the action of ROS and their derivatives in cultured cells are now available. For instance, ROS have been shown to activate signal transducing components, like p53 or members of the NF-κB pathway, resulting either in increased antioxidative protection or in activation of apoptotic pathways [2]. Nevertheless, a comprehensive integrated picture of the *in vivo *cellular responses of metazoans to oxidative stress is still not available. Genetic data suggest that different protection mechanisms are involved *in vivo *according to the type of ROS that induces the stress [4]. In addition, a wide diversity of macromolecules may undergo oxidative damage and induce secondary cellular stresses. These secondary effects of ROS could be similar to the alterations in macromolecules observed in other stress conditions, such as heat stress, endoplasmic reticulum (ER) stress (induced by accumulation of misfolded proteins), or UV-induced DNA damage. The relative importance of these common and specific responses to oxidative and other cellular stresses still has to be determined.

In the yeast *Saccharomyces cerevisiae*, microarray experiments have shown that similar transcriptional responses are observed in a large number of different environmental conditions, including oxidative stress induced by H_2_O_2 _or menadione [5,6]. According to the authors, this common environmental stress response (CER) may reflect the need for yeast to adapt quickly to rapidly changing external conditions. Similar transient variations of protein levels were also observed in proteomic experiments and highlighted the existence of an H_2_O_2 _stimulon [7]. It is not clear whether such common stress responses exist in long-living multicellular organisms since, unlike unicellular organisms, their cells are probably submitted to slower and smaller variations of the extracellular medium; furthermore, the survival of just one cell is not generally crucial for the survival of the organism as a whole.

Considering its powerful genetic and genomic tools, *Drosophila melanogaster *is a relevant model to address the question of the specificities of *in vivo *responses to various stresses in multicellular organisms and to identify novel genes that play a protective role. Nevertheless; there are some limitations to such studies on living flies. Firstly, adult flies are mainly composed of post mitotic cells; thus data obtained with flies may be relevant for comparison with the stress response of post mitotic tissues in other organisms (for instance mammals' neurons) but could be less useful to address the question of stress response in dividing cells. Secondly, limitations also arise from ROS-generating compounds delivery which in Drosophila is usually performed through food ingestion. This method severely limits kinetic studies of acute stress responses on flies, since, within a few hours, large fluctuations in the quantity of ingested food are observed in batches of flies transferred to a new medium. To overcome this problem some experiments were performed on starved flies. The major issue with such an approach is that the observed transcriptional changes could result from the starvation stimulus as well as from the effect of the studied compound. Therefore such experiments may in fact characterize the interference of two different stress responses with different induction times and kinetics rather than a *bona fide *oxidative stress response. A previous microarray study, performed with such a strategy on 4500 Drosophila genes, analyzed changes in transcription induced by paraquat, which produces superoxide radical (O_2_•^-^) intracellularly: 5.2% of the genes (n = 236) were found to be stress responsive. Kinetic analysis revealed that transcriptional modifications lead to the establishment of a more or less stable new expression profile 12 hours after stress induction, thus indicating the existence of a long term stress response (LTSR) [8]. This stability probably reflects the late response to paraquat-induced stress but variations before 12 h are more difficult to interpret since they may also arise from the stress effects of starvation. This analysis also did not address the question of the specific responses to different ROS and could not distinguish between specific responses to oxidative stress and responses common to other cellular stresses. Furthermore, since the arrays covered only 30% of the estimated total number of Drosophila genes, this study was also limited for functional statistical analysis and detailed analysis of functional classes involved in stress response.

From previous considerations, we chose to focus on comparisons of the long term transcriptional response (LTSR) of adult flies 24 h after ingestion of different stress-generating compounds. These responses may be representative of those of postmitotic tissue exposed to physiological chronic stress conditions (even if the level of stress is certainly higher in our experiments). Thus we investigated, at a full genome wide level, the transcriptional LTSR in adult Drosophila submitted during 24 h to three types of stresses: paraquat or H_2_O_2_-induced oxidative stresses and tunicamycin-induced ER stress. This latter drug inhibits N-linked glycosylation, thus leading to an accumulation of misfolded proteins in the ER (referred to as ER stress) which is known to elicit a specific response: the Unfolded Protein Response (UPR) [9]. We show in this paper that some of the transcriptional changes observed for these three stress conditions are similar, thus defining a class of multiple stress responsive genes. Nevertheless, in addition to this common long term stress response (CLTSR), many genes are transcriptionally regulated in a stress-specific manner. A statistical analysis identified classes of molecular functions or cellular processes over-represented inside clusters of genes undergoing transcriptional changes. Unexpectedly, both up and down regulations were observed for members of the same functional class. This may reveal novel functional specificities for these genes. In addition, we investigated whether genes that display significant transcriptional variations play a functional role in oxidative stress resistance. We present data suggesting that this could be the case for a large number of the stress-responsive genes identified in our study, which emphasizes the polygenicity of the stress responses, at both a molecular and a functional level.

## Methods

### Stocks

All the lines tested for paraquat stress resistance were collected from the Bloomington stock center. To minimize genetic background effects, when the mutation was linked to a *w*^+ ^transposon insertion, the line was outcrossed for 4 generations against a *w*^- ^isogenic strain of Canton S background before stress experiments. For homozygous lethal mutations we analyzed flies heterozygous for the mutation issued from a cross between the mutant line and the same *w *Canton S strain.

### Stress resistance tests and collection of fly tissues

We used 50 ml vials containing 1 ml of a solid medium composed of 1.3% low melting agarose, 1% sucrose and either 1% H_2_O_2_, 5 or 15 mM paraquat, 12 μM tunicamycin (all from Sigma) or no toxic compound (control tubes). These compounds were incorporated at 45°C to avoid loss of activity.

3 day old males were placed in groups of 30 in these vials and maintained at 26°C with a 12:12 light-darkness alternation.

In survival tests, dead flies were counted twice a day until the end of the experiment. In each experiment at least 3 vials of 30 male flies were used. To test mutant lines we performed three independent experiments in order to minimize false positive detection. Survival data were submitted to a log-rank analysis to detect statistically significant survival differences between mutant and *w *Canton S flies. We considered that a mutation had a significant effect on survival under oxidative stress when the mean of log_10 _(p-log-rank) for the three experiments was lower than -3 and at least two experiments had p-log-rank < 0.001. For microarray experiments, for each condition, 200 Canton S males were kept 24 hours on the corresponding medium and then frozen in liquid nitrogen for subsequent RNA extraction. Independent batches of males from separate experiments were used for replicate experiments. All fly manipulations were performed at the same stages of the 12:12 light cycle to prevent any undesirable effects from circadian variations.

### Sample preparation and data analysis

We analyzed 4 samples of control flies and 3 (paraquat 15 mM) or 2 (all other conditions) samples of stressed flies. Total RNAs were purified by three rounds of Trizol reagent (GIBCO/BRL) extraction before precipitation. cDNA were synthesized from 10 μg total RNA aliquots and biotin-labelled cRNA targets synthesized using the BioArray high yield RNA transcript-labelling kit (Enzo Biochem) according to the manufacturer's instructions. Hybridizations on Drosophila Genome Arrays (Affymetrix) and subsequent washing were performed on a GeneChip Fluidics Station according to the manufacturer's instructions before scanning on a GeneArray scanner.

Data extraction was performed first by the MAS5 Affymetrix program which provides absolute values (AV) and detection *p-values *(DP) for each probe set. These data were loaded into an Access database for subsequent analysis. We retained only the experimental points that presented a mean value greater than 0.1 for the DPs of all the different samples of at least 1 of our 4 experimental conditions. This reduced the number of probe sets further analyzed from 14028 to 8976. The AV data from each microarray were then normalized against the AV mean value of the 4 control samples by a quantile method which performs optimally [10]. Since a large number of flies (~300) were used for each RNA sample hybridized to a microarray, variations in signal arising from individual transcription differences are greatly reduced. This is reflected in the high values of correlation coefficients between microarrays corresponding to the same experimental condition (data not shown). The normalized values were used for further comparison of each of the stress condition samples with the 4 control samples and for statistical validations of the variations using the SAM program [11]. For this analysis, we used a fold change threshold value of 1.5 and a mean FDR (false detection rate) lower than 10%. 1368 independent probe sets that fulfilled these conditions for at least one type of stress were retained for further analysis.

A hierarchical divisive clustering of the data of these probe sets was performed using the SOTA [12] implementation available at . For each probe set, the ratios for all the combinations between stress conditions AVs and reference AVs were computed and Ln2 transformed. The SOTA algorithm used on this dataset with linear correlation distance with 0 offset, 1000 cycles and 1.01 variability threshold parameters, led to the detection of 19 clusters.

### Functional analysis

Information from the Gene Ontology (GO) database (December 2003, [13]) was combined with the Affymetrix data through the THEA program  to investigate which classes are over- or under-represented in the dataset of stress responsive genes. Briefly, according to the Gene Ontology hierarchical structure, each probe set was assigned, when possible, to its original annotation and to the associated parent annotations. The number of probe sets for the different GO terms was computed for groups of probe sets defined according to different criteria (such as whole microarray probe sets, detected probe sets or probe sets belonging to a given cluster).

For each GO term G, the distribution between the group D of all the detected probe sets (N^G^_D _probe sets issued from a total of N_D_, probability P_G _= N^G^_D_/N_D_) and a group C of particular interest, such as a cluster (N^G^_C _probe sets issued from a total of N_C_) are compared. The hypothesis of equal repartition between these two groups would predict that, inside the N_C _probe sets of group C, N_C_*P_G _probe sets should be associated to the GO term. We computed the *p-value *P_N _for the null hypothesis of no association between the two distributions, with a binomial distribution with N_C _tries, a probability P_G _and N^G^_C _successes. Threshold values for P_N _helped to define the GO terms over- or under-represented in the group C.

### Quantitative real time RT-PCR analyses

Experiments were performed as described [14] with 2 μg of the total RNA samples used for microarrays (control, P15, P5, H1 and T12). Primers were designed to generate an amplicon of about 100 nucleotides and their sequences are described below (Forward/Reverse primers):

FBgn0015039: TATGCTCTTCAACCTACTGCTGC/TAGGCGTAAAATTGAATCCACTC

FBgn0010383: GACGCTGAACGGATATGGCAT/ATGTAGGTCATCCCGAACTGTC

FBgn0015035: CAACTCTGAATTTGGCTCTCATCC/AGCGGGTTTCTCCTCCTCAA

FBgn0034334: GAAGCCGGATATGTTACGCAAG/TTCACCAGATAGCCGATGATG

FBgn0038024: CCTCAACAAGTACCCGAATGTG/TACTCCCTTCAGTTCCACGGC

RP49: CCGCTTCAAGGGACAGTATCTG/CACGTTGTGCACCAGGAACTT

Annealing temperature was 62°C except for RP49 and FBgn0034334 transcript level quantifications, for which it was 60°C. We normalized samples by comparison with the levels of the RP49 housekeeping gene. Levels of transcripts under various stress conditions are compared with the transcript level observed in control flies.

## Results

### Transcriptome variations in adult Drosophila are strongly dependent on the type of stress to which they are submitted

**Figure 1:**
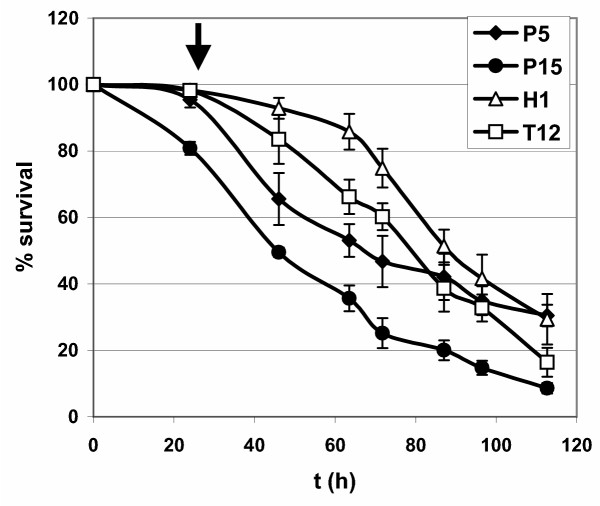
**Lifespan reduction in *Drosophila *submitted to paraquat-, H_2_O_2_- and tunicamycin-induced stress **3-5 day-old Canton S wild type males were placed at t = 0 by groups of 30 in vials containing 15mM paraquat (P15: ●), 5 mM paraquat (P5: ◆), 1% H_2_O_2 _(H1: △) or 12 μM tunicamycin (T12: □). Dead flies were counted twice a day to determine survival. 3 vials of 30 individuals were used for each condition. When no toxic compound had been incorporated in the medium, more than 90% survival was observed at t = 120 h (not shown). Similar average lifespan was observed for P5, H1 and T12 around t = 80 h while it was significantly reduced in P15. Arrow indicates the time (t = 24 h) at which flies were collected for RNA extraction. Note the 20% lethality observed at this time for P15 condition. Dead flies were discarded before RNA extraction.

We wished to compare the transcriptomes of flies submitted to continuous stresses induced by ingestion of paraquat, H_2_O_2 _or tunicamycin at concentrations leading to similar effects on viability. Survival curves were obtained for 3 day old male flies raised on media containing different concentrations of these drugs (Fig. 1). Concentrations of 1% H_2_O_2_, 5 mM paraquat and 12 μM tunicamycin had similar effects on the survival of flies and were chosen for further studies. A paraquat concentration of 15 mM was also used for comparison with previous studies [8].

RNA were obtained from separate experiments with 3 day old male flies reared at 26°C with a 12:12 hours light and dark (LD) alternation, on media containing no drug (4 reference samples), 15 mM paraquat (P15: 3 samples), 5 mM paraquat (P5: 2 samples), 1% H_2_O_2 _(H1: 2 samples) or 12 μM tunicamycin (T12: 2 samples). Thus a minimum of 8 pairwise comparisons were made for each condition which ensured good statistical significance, as confirmed by quantitative PCR experiments (see below). Stresses were induced 24 h before collection of flies, which occurred at the same time (9 h) of the 12:12 hours light/dark cycle to eliminate the effect of circadian variations. Hybridizations were performed on Affymetrix GeneChips and the data processed as described in the Material and Methods. The statistical significance of transcriptional variations was assessed using the SAM program with a threshold of 1.5 [11]. A good correlation was observed between our P15 results and previous studies with the same stress conditions [8]: among the 246 stress responsive ESTs of Zou *et al.*, 201 were associated to a detectable probe set on our chip, 56% of which were selected by SAM analysis and 72% of which displayed a fold change greater than 1.3 (not shown). The remaining discrepancies may arise from differences in statistical selections, in analyzed tissues (thorax and abdomen in [8], whole flies in this study) or in genotype: compared to the *w*^1118 ^flies used in [8], our wild type Canton S flies were more resistant to paraquat 15 mM (mortality of 20% *vs *54% at 24 h) and presented an increased medium lifespan (48 *vs *35 days at 26°C, on standard medium).

Among the 8976 probe sets significantly detected in adult flies (see Material and Methods), 1111 were up or downregulated with P15 treatment, this number being reduced to 608, 72 and 221 for P5, H1 and T12 treatments respectively. Thus, even with similar effects on flies survival, the fraction of the genome detected as stress responsive on microarrays was highly dependent on the nature of the stress, varying about ten times from 7% (P5) to 0.7% (H1). This first analysis defined a total of 1368 probe sets and 1343 genes which are induced or repressed at least in one stress condition. They were used for further analysis.

### Common and specific responses to different stress

**Figure 2:**
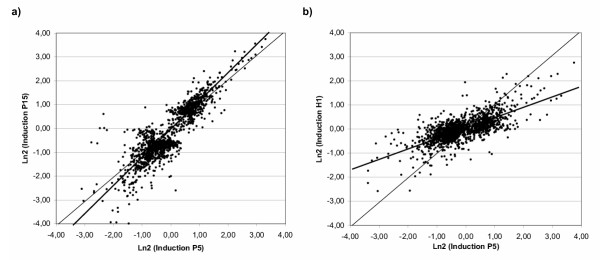
**Correlations between P5 and P15 or H1 microarray measurements **For each of the 1368 probe sets selected in the SAM analysis, the mean Ln2 ratios between the absolute values (AV) for stress and reference conditions were compared in two dimensional plots. Bold lines are the linear regression curves for the two comparisons, the thin lines correspond to a complete correlation for eye guidance. A good correlation is observed between P5 and P15 with a slope of 1.14, while it is much weaker between P5 and H1 (slope of 0.4).

We plotted transcriptional variation correlations for the different oxidative stress conditions (Fig. 2). We observed a high degree of correlation between the two paraquat experiments (correlation coefficient c = 0.86, Fig. 2a). The slope of the linear regression curve, however, was 1.14 which indicates that variations in transcription induced by paraquat may be dose-dependent for most genes in *D. melanogaster*. Lower correlations were observed for the linear regressions between P5 ratios and either H1 ratios (c = 0.64, slope = 0.40, Fig. 2b), or T12 ratios (c = 0.43, not shown).

Clustering analysis provided further information about the specificity of stress responses. We chose an unsupervised divisive clustering method (SOTA [12]) to analyze the data and we checked that other methods such as Self Organizing Maps [15] yielded similar results (not shown). The SOTA analysis predicted 19 clusters. The complete list of the 1368 probe sets with their cluster assignment is provided as Tab.S1 (Additional file1) in supplementary data. In Tab.1, (Additional file 8) we present the average log-ratios in each stress condition for the 19 identified clusters. These data confirm the high correlation between the results for P15 and P5 and the general tendency toward smaller variations for P5. However, the genes included in cluster 7 exhibit a more severe repression in the P5 condition than in the P15 condition which may reflect a differential transcriptional response as a function of oxidant concentration.

Notably, in clusters 5, 6, 7, 9, 10, 13, 16 and 18 which regroup 642 probe sets, significant variations for H1 were observed in the same direction than for P5 or P15. This suggests that, for a large number of genes, both oxidative compounds induce similar transcriptional responses. Therefore, the fact that the number of probe sets validated by the SAM procedure as being significatively affected in the H1 condition is smaller than in the paraquat conditions may be a consequence of a similar but weaker effect of H_2_O_2 _on the transcriptome rather than fundamental differences in the responses to the two oxidants.

What is the specificity of the oxidative stress responses induced by paraquat or H_2_O_2 _compared to the ER stress response induced by tunicamycin? The 19 clusters from Tab.1 (Additional file 8) can be regrouped into 7 large classes of genes: Classes A and B contain genes respectively downregulated and upregulated in both oxidative stress and ER stress conditions. Inside these two large groups, 237 genes included in clusters 9, 10 and 13 are regulated in a similar fashion in all four stress conditions. Genes from classes C and D (48% of stress responsive probes) are respectively downregulated and upregulated by oxidative but not ER stress. Conversely, class F genes are upregulated in ER stress but not in oxidative stresses. In the atypical classes E and G, opposite variations are observed for the two types of stress: genes of class G are upregulated by ER stress but downregulated in oxidative stress while genes of class E display an opposite behavior. Overall, our data emphasize both specificities and similarities in these stress responses: the classes A and B (238 and 276 probe sets, respectively) which include genes displaying similar responses to both oxidative and ER stresses, represent a sizeable fraction (38%) of the stress responsive probes. In contrast, genes that vary in opposite directions, included in the classes E (104 probe sets) and G (60 probe sets), represent a smaller part of those stress responsive probes (12%).

### Classes of stress responsive genes

Using the Gene Ontology annotation [13] we identified the molecular functions that are over- or under-represented among all the 1368 stress-responsive probesets compared to the distribution of functions identified for the complete set of 8976 detectable probesets (see Material and Methods). The analysis was first performed independently for each set of genes validated by the SAM procedure for each stress Table 2a to c (Additional file 9). A similar analysis for biological processes is given in supplementary Table S2 (Additional file 2). The most prominent functional classes over-represented in the paraquat sets are the peptidases (including peptidases which are part of the proteasome complex), the peptidase inhibitors, the glutathione transferases (GT) and oxidoreductase enzymes or electron transporters, including the P450 cytochromes. These classes could all be involved in the detoxifying processes that follow oxidative stress and are discussed in more detail below. In addition, lipases and more prominently the triacylglycerol lipases, also over-represented, may contribute to the regeneration of membranes after oxidative damage. Most of these features seem to be part of a general stress response since triacylglycerol lipases, peptidases with chymotrypsin or trypsin activity and GTs are also over-represented in the H_2_O_2 _and tunicamycin specific sets of genes. The transaminases, the cyclohydrolases, the oxidoreductases and the hydroxymethyltransferases define the signature of functional classes over-represented in the two types of oxidative stresses. In contrast, proteins which bind to iron ions or monooxygenases are specifically over-represented in the paraquat set. As expected, the ER set presents features that are distinct from oxidative stress responses, that is the over-representation of hydrolases acting on glycosyl compounds, UDP-glucuronosyltransferases and tRNA ligases. This last class suggests that modifications of the translation rate may be an *in vivo *response to ER stress. Besides these ER stress-specific classes, peptidases with elastase activity and epoxide hydrolases are over-represented in both paraquat- and tunicamycin-induced stresses. Interestingly, this last class of proteins is involved in the metabolism of juvenile hormone which has been shown to be involved in heat stress response [16].

We then performed a similar analysis for the groups of genes identified in the clustering process. To increase the statistical significance of the analysis, we used the 7 groups A to G instead of the 19 initial clusters. This analysis, given as Tables S3 (Additional file 3) and S4 (Additional file 4) of supplementary data, allowed us to identify molecular function and process signatures in some clusters. For instance, for the genes repressed for oxidative and ER stress conditions (group A) specific over-representations are observed for alkaline phosphatases, diazepam binding proteins and proteins involved in acyl-CoA metabolism. Signatures of group B (genes upregulated for oxidative and ER stresses) include proteins involved in response to abiotic stimuli, including GTs and glutathione peroxydases, and tRNA ligases. This last feature may indicate that the organism reacts to sustained stress by an increase of protein synthesis. Nevertheless, genes involved in proteins biosynthesis are surprisingly under-represented among the stress responsive genes. Retinoid binding proteins and transporters are specifically over-represented in group C. Surprisingly, in this group of genes, a large number of peptidases are present along with a strong proportion of protease inhibitors. Signatures for group D (genes upregulated under oxidative but not ER stress) include chaperones associated with the heat shock response, glutamate synthases and proteins involved in ATP-dependent proteolysis. Glutamate synthases, together with the upregulated genes *Ahcy13 *and *Eip55E*, may be required to increase the pool of glutathione, a major actor in redox regulation and phase II detoxification [17]. Additional signatures for group D include two other processes, inosinate (IMP) biosynthesis and amino acid biosynthesis. Interestingly, we found under-representation of their parent processes (closer to the root of the ontology), namely nucleic acid metabolism and protein biosynthesis. Finally, in the ER stress specific groups F and G, the disulfide isomerase proteins and the glucuronosyltransferases, known to play an important role in the UPR following ER stress in yeast, are over-represented together with proteins involved in lipid metabolism.

Overall, our data suggest that oxidative and ER stress induce comparable transcriptional modifications of a significant number of genes known to be involved in a limited number of functional classes.

### Gene-specific stress responses inside functional classes

In contrast to previous work limited to partial analysis of the genome, the use of whole genome Affymetrix chips allowed us to investigate the specificity of transcriptional responses for genes associated with a given functional class.

The thioredoxin system plays a major role in oxidative stress defense and needs to be better functionally characterized. In Drosophila, the peroxiredoxin proteins show thiol-dependent peroxidase activity and use thioredoxin, but not glutathione, as a source of reducing power. Indeed, Drosophila lacks glutathione reductase [18] and its function is apparently substituted by thioredoxin reductase. Interestingly, we observed significant differences in the transcriptional behavior of the members of the thioredoxin system when flies were submitted to paraquat stress. The thioredoxin class (GO:0030508) counts 7 members with either a sequence matching perfectly the consensus catalytic site WCGPCK (*CG4193*, *CG3864*, *Txl/CG5495 *and *CG1141*) or with one mismatch (*CG8993*, *CG13473 *and *CG3719*). Only the *Txl *gene is significantly overexpressed over the 1.5 fold threshold, the other genes presenting no change or a weaker overexpression (*Trx-2*). This strongly argues for a specificity of these thioredoxins in the defense process with an important role for the *Txl *gene. Similarly, among the five genes presenting a thioredoxin peroxydase activity (GO:0008379), only two (*CG12013 *and *CG1633*) are overexpressed, the others (*CG12174*, *CG5826 *and *CG6888*) being unaffected in the studied conditions. Among the related genes only the peroxyredoxin *CG11765 *is overexpressed, while the glutathione peroxydase-like *CG15116*, very similar to the thioredoxin peroxydase *CG12013*, is significantly repressed. These specificities strengthen the concept of a functional diversification of these proteins in spite of their common ability to confer resistance to oxidants in Drosophila cells [19].

When the organism is challenged to oxidative stress, in addition to performing direct enzymatic detoxification of toxic compounds, it must also limit the appearance of the most toxic species. Therefore, since free iron catalyses the production of the highly toxic hydroxyl radical (OH•) from H_2_O_2 _by the Fenton reaction, its concentration must be tightly controlled. Transferrin and ferritin proteins play a major role in this control [20]. Furthermore, variations in iron concentration may modify gene expression in the cell through the iron regulatory proteins Irp that bind to the iron responsive elements (IRE) located in their target genes UTRs. Under paraquat stress, we observed a coordinated and specific response of genes used in regulation of free iron concentration and iron-regulated response: the two ferritin subunits and the iron regulatory protein 1B (*irp1B*) are overexpressed, while the transferrin 1 (*tsf1*) gene is severely repressed. Nevertheless, neither the *irp1A *nor the *tsf2 *and *tsf3 *genes show any significant transcriptional change. This suggests that each isoform of these families plays a specific role in iron homeostasis in the organism.

More complex specificities can be observed in larger functional classes. The glutathione transferases (GTs; GO:0004364) play important roles the detoxification process after genotoxic stresses [21]. As expected, a large number of them (16/34) are overexpressed after paraquat-induced oxidative stress Table 3a (Additional file 10) but 4 are underexpressed under the same conditions. One of these GT repressed by paraquat (FBgn0034334) is also severely repressed by H_2_O_2_-induced stress. Moreover, among the 16 GTs overexpressed in paraquat-induced stresses, 7 are overexpressed and 3 underexpressed in ER-stressed flies, while 6 show no other significant transcriptional variation. Interestingly, all the GTs overexpressed in both paraquat and tunicamycin experiments are also slightly induced in H_2_O_2_-stressed flies. Overall, our data suggest that both "generalist" GTs that are able to protect the organism against various stresses and more specialized GTs, required only for protection against well defined stresses, coexist inside the cell.

**Figure 3:**
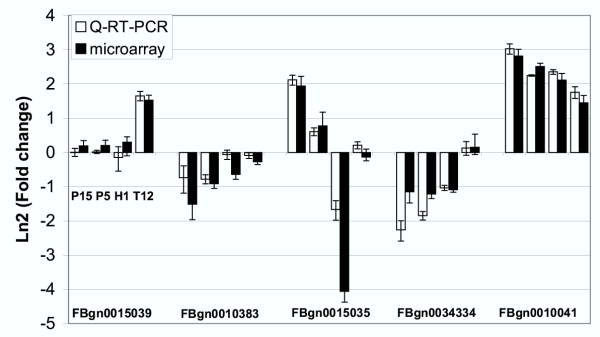
**Comparison of transcript level variations detected with microarrays and with quantitative real-time PCR (Q-RT-PCR) **Transcript levels were analyzed for genes encoding three P450 cytochromes (FBgn0015039, FBgn0010383 and FBgn0015035) and two glutathione transferases (FBgn0034334 and FBgn0010041). The Ln2 ratios between the transcript levels under stress conditions (P15, P5, H1 and T12) and the reference condition, obtained with Q-RT-PCR (white bars) and microarray analysis (black bars), are indicated for each gene. Error bars: standard errors.

A similar conclusion can be drawn for the P450 cytochromes (GO:0015034). Among 58 detectable P450 cytochromes, 12 are underexpressed and 12 overexpressed during paraquat stress, 4 of these latter being also upregulated in tunicamycin-stressed flies (Tab.3b, Additional file 10). We observed a general tendency of these paraquat-inducible P450 cytochromes to be also overexpressed in H_2_O_2_-stressed flies. One cytochrome gene (FBgn0015035) displays peculiar behavior since it is induced by paraquat but strongly repressed by H_2_O_2_. Another gene (FBgn0015039) is induced specifically by tunicamicyn. Quantitative RT-PCR experiments confirmed the specificities observed on microarrays (Fig. 3).

The complete data for the peptidases class (GO:0008233) analysis – given as Tab.S5 (Additional file 5) of supplementary data- provides a striking feature: most of the 131 peptidases selected by the SAM analysis (among 361 that were detectable) are downregulated by either both paraquat-induced oxidative stress and ER stress (54 peptidases) or paraquat-induced stress only (41 peptidases); nevertheless, a small number (36) of them are upregulated by paraquat. Closer examination of these latter genes revealed that 22 are proteasome endopeptidases. Further analysis of the proteins belonging to the proteasome complex, (GO:0000502) (which also contains proteasome regulatory proteins) shows that 33 out of 45 detectable proteasome constituents (73%) are likely upregulated by paraquat treatment (Tab. 3c, Additional file 10), both 19S and 20S subunits being coordinately regulated. Interestingly, the induction level is clearly correlated to the dose of paraquat used. Moreover, this induction is very specific since it is not observed in H1 or T12 conditions for any of these genes. The functional significance of this observation needs to be addressed in Drosophila strains mutant for proteasome subunits, challenged with paraquat, H_2_O_2 _or tunicamycin stresses.

### Many genes transcriptionally affected by oxidative stress modulate oxidative stress resistance

**Figure 4:**
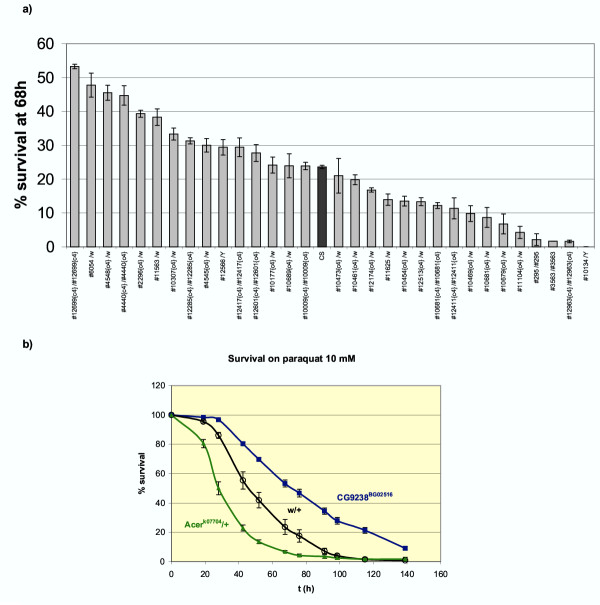
**Resistance to paraquat-induced stress of flies mutant for genes identified in microarray experiments **a) 29 Drosophila lines bearing mutations in genes identified in our microarray experiments as being stress-responsive were recovered from public stock centers. When the mutation was linked to a *w*^+ ^transposon insertion these lines were outcrossed with a *w*^+ ^Canton S reference line. 3–6 day old male flies were then tested for their resistance to oxidative stress 68 h after transfer to 10 mM paraquat medium. Tested flies were either homozygous (notation #i/#i in the X axis) for viable mutations or heterozygous (notation #i/*w*) for lethal mutations (in this case they are issued from a cross with *w*^+ ^Canton S females). For simplicity, identification of lines (#i) refers to the Bloomington stock number and the genotype of the line is provided in Tab. 4. We present in this Figure the results of one of three independent experiments that we used for the complete statistical analysis presented in Table 4. Compared to male flies issued from a cross between *w*^- ^males and Canton S females (noted w/+, dark bar), significant differences in resistance or sensitivity to paraquat can be observed for a large number of the lines tested. Error bars: standard error. b) Example of survival curves on 10 mM paraquat-containing medium of some mutant male flies. Flies heterozygous for a lethal mutation in the Angiotensin converting enzyme related (*Acer*) gene are sensitive to paraquat, while flies homozygous for an insertion in the gene *CG9238 *are clearly more resistant to paraquat than *w*/+ control flies. Neither of these genes was previously suspected to play a role in oxidative stress resistance.

When a fly experiences an oxidative stress we can expect that the subsequent transcriptional modifications may arise from several mechanisms. Firstly, the organism can mount a protective response, for instance by inducing proteins which will reduce adverse consequences of the toxic compound. Only a few functional classes (such as GTs, electron transporters, chaperones) identified in our functional analysis of stress-regulated genes can be clearly associated to such known protective mechanisms from oxidative stress (Tab. 2, Additional file 9). Secondly the toxic drug itself may induce transcriptional changes which could play a role in its toxicity. The relative part of these protective or toxic responses to oxidative stress is unknown. We thus investigated whether genes detected in our microarray analysis could be involved in oxidative stress protection against paraquat or in its induced toxicity. We addressed this issue using a genetic approach, taking advantage of the availability of numerous strains bearing mutations in genes detected in the microarray paraquat set. Twenty nine such lines were recovered from public stock centers and adult flies were analyzed for their survival after transfer to a medium containing 10 mM paraquat. Most of the mutations used arise from P elements insertion in the 5' regulatory region of the genes which are expected to induce partial or complete loss of function mutations. Indeed, as shown in table 4 , most of them have been characterized as either lethal recessive mutations or hypomorphic loss of function mutations and, in some cases, do not complement a deficiency. Particular attention was paid to ensure that the genetic background was controlled in these experiments and stringent statistical conditions were used for the data analysis (see material and methods). Several conclusions can be drawn from these genetic experiments. a) First, as shown in Fig. 4a, under these conditions, a high proportion of the 29 tested strains present statistically significant survival differences from the *w *Canton S reference strain. Indeed, the results of our experiments show that 13 mutant lines out of 29 tested (45%) are either significantly more resistant (6 lines) or more sensitive (7 lines) to paraquat than their wild-type counterparts (Tab. 4, Additional file 11 and Fig. 4b). This ratio is at least 10 times higher that what is expected from previous genetic screens (see discussion) and suggest a strong relationship between transcriptional stress response and functional *in vivo *susceptibility to oxidative stress. b) For the genes studied there is no clear correlation between the observed induction or repression under paraquat treatment and the effect of the mutation on the paraquat resistance or sensitivity phenotypes (Tab.4, Additional file 11). This suggests that, in the steady stress conditions used, both deleterious and protective gene regulations are taking place. c) Our genetic data point out the large functional diversity of genes that are able to modulate the oxidative stress resistance *in vivo*: ion channel (*Sh*), thioredoxin reductase (*Trxr-1*), fatty acid elongase (*Baldspot*), phosphatase (*aay*) and phosphatase regulator (*CG9238*), transcription factor (*Xbp1*) and peptidase (*Acer*). Interestingly, among these 13 mutants, only 2 were previously known to be associated to oxidative stress resistance (*Sh *and *Trxr-1*) and most of them had no known function in adult flies. Coupling between microarray and genetic experiments is thus a powerful way to extend our knowledge on the biological function of Drosophila genes without biased hypothesis and to provide some clues on the function of mammalian homologues.

For instance, we found that the *Dgp-1 *gene is induced in flies challenged with paraquat stress and that its disruption leads to stress resistance. The Dgp-1 protein is strongly similar to the mammalian GTPBP1 protein which presents a GTP binding domain and strong similarity with the elongation factor Ef-Tu [22]. Interestingly, expression of GTPBB1 is enhanced by gamma interferon in a monocytic cell line, suggesting that this protein in involved in host defense mechanisms. Nevertheless, no phenotype was observed in mice disrupted for this gene, maybe because of compensation by a gene of the same family [22]. Our data provide evidence that, in flies, Dgp-1, the GTPB1 homologue, is indeed involved in protective mechanisms against stress. The similarity with EF-Tu suggests that this protection might be linked to a downregulation of protein synthesis. In agreement to this hypothesis, it is noticeable that mutants for the translation negative regulator Thor present a significant sensitivity to paraquat stress (confidence index -2,4 in Tab. 4, Additional file 11 and Fig. 4a) and has been shown to be sensitive to bacterial infection [23].

## Discussion

In this paper we present the characterization of the *in vivo *transcriptional responses of adult Drosophila males submitted to four different continuous stresses : paraquat (two conditions), H_2_O_2 _or tunicamycin. Experiments on yeast submitted to several types of stress including oxidative stress have shown that fast transient responses occurring during the first three hours are followed by stable long term (>12 hours) changes [5,6]. Similarly, previous experiments on paraquat-induced stress in Drosophila have shown sustained long term changes in transcript levels which are more or less stable 12 hours after stress induction [8]. Since, as discussed previously, there are clear technical limitations to short term kinetic studies on Drosophila submitted to ingestion driven stress, we focused our efforts on the observation of these long term stress responses (LTSR) and performed our transcriptome analysis 24 hours after stress induction. At this time point, more than 95% of flies were alive for P5, H1 and T12 treatments, while 19% of lethality was observed in the P15 experiments. In addition, during the next 24 hours, in all conditions, less than 30% of the animals died. We thus expect that any secondary effects linked to the level of lethality are minimal in our experiments. In agreement with this assumption we noticed that in the experiments of Zou *et al. *similar results were obtained when the transcriptome was analyzed 12 hours (when lethality was negligible) or 24 hours after ingestion of 15 mM paraquat. Furthermore, when functional analysis was performed, we were unable to detect significant differences in the signature of the genes detected in the P5 and P15 experiments, which should be the case if the level of lethality plays an important role for gene transcription. We thus conclude that the secondary effects linked to the levels of lethality in the Zou et al. experiments and in our work do not significantly affect the transcriptome and that the variations observed are primarily due to the stresses experienced by the flies.

Our data present clear evidence of a common long-term stress response (CLTSR) in transcription of Drosophila genes: at least 237 genes contained in clusters 9, 10 and 13 show similar changes in transcription for the three stressors studied. This number could be a minimum estimation of the extent of the CLTSR, since it is mainly limited by the weaker transcriptional variations observed in the H_2_O_2_-induced flies. We think that this may be due to a smaller number of cells experiencing stress when flies ingest H_2_O_2_. Additional data for comparison with various stress responses (immune stress [24], starvation [25] and, during the submission of this work, hyperoxia and aging [26]) are presented in Supplementary text T1 (Additional file 7) and Table S6 (Additional file 6) and confirm the existence of a core of similar transcriptional responses between these stresses.

The CLTSR shows certain similarities with the common environmental response (CER) described in yeast [5,6]: in both cases heat-shock genes, genes involved in the detoxification processes, or associated with fatty acid metabolism and DNA repair show similar changes in all the stress conditions studied. Nevertheless, there are also obvious differences between these two responses. For instance, in contrast to what occurs in CER, no large scale coordinated transcriptional changes for genes involved in translation inhibition or energy production were detected in CLTSR. This may reflect the fact that, in our experiments, the CLTSR corresponds to a long-term adaptation of the stressed Drosophila cells, while the variations observed in yeast are transient (of course we cannot exclude long term post-transcriptional modifications in the translation apparatus and the metabolic pathways activities of stressed flies). Alternatively, these data may reflect differences in the adaptation of dividing cells (yeast) and post-mitotic cells (Drosophila) to stress conditions. For instance, in the latter case, upregulation of the iron responsive protein 1b gene may lead to translational downregulation of the succinate dehydrogenase gene through an IRE [27] and hence modulate energy production as in the yeast, but in a different way. Additionally, in Drosophila, translation repression may also be involved in stress response but relying on a small subset of genes (which would then not have been detected with our functional analysis). Interestingly, in support to this hypothesis, we found that the translational repressor *Thor *is induced under stress conditions and that mutations in this gene confer a slight but significant sensitivity to paraquat-induced stress. However, our finding that tRNA ligases are upregulated in oxidative and ER stress may indicate a requirement for increased protein synthesis under sustained stress conditions. Kinetic studies using another oxidative stress paradigm are needed to clarify this point. In view of our results, it would be also interesting to investigate possible variations in stress response in mammalian tissues either mitoticaly active or quiescent.

Besides their similarities, the LTSRs also display marked differences. One of the most striking specific expressions is displayed by the genes encoding for the proteasome subunits. These proteins belong to the two large complexes 19S (regulatory complex) and 20S (proteolytically active complex) which, together, form the 26S proteasome [28]. Most of them (73%) are specifically induced by paraquat- but none by H_2_O_2_- or tunicamycin-induced stresses. It is also noticeable that, in contrast to proteasome constituents, ubiquitin protein ligases are under-represented among paraquat responsive genes. The 20S proteasome, inactive in its native form, is able to specifically degrade oxidized proteins *in vitro *and *in vivo, *and has been considered to be the main actor in this process [29]. Nevertheless, it has been recently proposed that, while the 20S proteasome is active during oxidative stress and limits the accumulation of oxidized proteins, the 26S, inactive in presence of ROS, "cleans" the cell in the following recovery process, eliminating thereby the accumulated altered proteins [30]. This seems to be a very important aspect of oxidative stress defense since oxidization of proteins can result in protein fragmentation and partial unfolding, and induce the formation of cytotoxic insoluble aggregates, a process that is known to be implicated in an increasing number of human pathologies [31,32]. The observed coordinated upregulation of genes encoding both 19S and 20S proteasome subunits when Drosophila cells are submitted to continuous paraquat stress strongly suggests that both complexes are indeed important *in vivo *for oxidized proteins degradation.

We observed no such induction of proteasome components in H_2_O_2_-stressed Drosophila. This result is coherent with previous studies shoving that the proteasome subunit are not transcriptionally regulated in cultured mammalian cells treated with H_2_O_2 _[33]. However this is surprising since it has been shown, in mammalian cells, that the proteasome is in fact involved in the degradation of misfolded glycoproteins as well as oxidized proteins after H_2_O_2 _treatment [34]. Recent data in lens epithelial cells showed that H_2_O_2 _induces an increase in proteasome activity and E1 ubiquiting activation enzyme levels without any increase in E1 mRNA levels [35]. In view of our data, we propose that two different strategies are used in *D. melanogaster *to deal with oxidative challenge and increase proteasome activity: one response, induced by H_2_O_2_, would rely on post-transcriptional mechanisms as shown in mammalian cells; while the other response, induced by paraquat, would rely on coordinated increase of transcription of the proteasome genes of both 19S and 20S subunits.

A number of functional classes are clearly over-represented among the genes involved in the LTSRs. The analysis of these specific functional classes revealed an important heterogeneity of stress-specific responses among their members. For instance, we have shown that only a subset of genes potentially involved in the thioredoxin pathway are upregulated during paraquat stress. Whether the remaining genes are involved in an earlier phase of the stress response, in a subset of tissues or in other processes unrelated to stress protection needs to be addressed. Interestingly, in agreement with this last hypothesis, one of these genes, *Jafrac2, *which codes for a thioredoxin peroxidase, has been recently assigned an unexpected role in caspase-regulated cell death [36]. The P450 cytochromes and the glutathione transferases also display striking stress-specific responses. For the GTs, 3 genes are downregulated by tunicamycin and 4 by paraquat, while 6 are upregulated by paraquat and 7 by both drugs. When we tried to correlate this information with GT classifications [21] we found that the latter group contained almost exclusively δ-type GTs (Table 3a). This suggests that this insect-specific class, unlike other Drosophila GTs, may have acquired a broad-spectrum detoxifying function which is required to counteract both oxidative and tunicamycin-induced cellular damages and/or that these GTs molecular targets are altered in both types of stress.

One important issue is whether our findings are representative of long term transcriptional responses in Drosophila submitted to real physiological chronic stresses. Indeed, the stress levels experienced by flies in this work are probably much higher than those experienced in real life. Nevertheless, the tight correlation that we observe between P5 and P15 experiments demonstrates that most of the genes undergoing transcriptional changes at a high concentration of paraquat display similar changes (although at a reduced level) when the concentration is threefold lower. This suggests that many genes identified in this study may also be induced in low intensity chronic stress.

A striking feature of our results is the large number of genes not previously associated with stress response which show transcriptional changes under paraquat-induced oxidative stress conditions. We investigated the biological validity of these observations in a genetic study of mutations in some of these genes. Since our microarray data suggest that the stress responses may be highly polygenic (with at least 10% of the genome involved), we took a particular care to ensure that there was a controlled genetic background in these experiments. We found that 45% of the mutations tested were associated with either resistance or sensitivity to paraquat, which confirms this idea of a highly polygenic process. It should be stressed that, since many of the tests were performed on heterozygous flies, the proportion of genes functionally involved in oxidative stress resistance may be higher. Extrapolation of the results obtained with this small subset of 29 genes to the 1107 genes found to be regulated by paraquat, suggests that some 500 genes may modulate paraquat sensitivity *in vivo*. This contrasts with two previous genetic screens to detect paraquat hypersensitive mutants, which concluded that only a few genes are involved in paraquat hypersensitivity [37,38]. These studies however analyzed only EMS viable mutations on the X, 2nd and 3rd chromosome. They would thus have missed any lethal mutations that could confer a sensitivity phenotype to heterozygous flies by gene dosage reduction. In fact, when we performed a P{*w*^+^; UAS}- based screen we found that a large proportion of P-element insertions may confer H_2_O_2 _or paraquat resistance or sensitivity ([14] and Girardot et al. unpublished) in agreement with the results presented here.

If all the transcriptional responses to a stress were protective for the organism; we would expect a clear correlation between the direction of the transcriptional response of the genes studied and the effect of their mutations on stress resistance. A significant result of our experiments is that we could not find such a correlation.

It thus appears that the transcriptional responses to oxidative stress may be either protective or deleterious for the flies. The simplest explanation for this result is that, besides the protective responses mounted by the organism cells (for instance in inducing detoxifying proteins), the paraquat also induces transcriptional changes that play a role in its toxicity. In mammalian cells, several transcription factors may be regulated by oxidative stress, either by direct modification by the ROS or through signaling pathways, and have either pro- (Jun, p53) or anti-apoptotic effects (NF-κB, HSF1) [2]. In addition, the choice between survival and apoptosis may depend on the intensity of the stress and on the cell type, as it has been clearly demonstrated in the case of p53 [39]. Signaling pathways which activate these factors are strongly conserved between mammals and Drosophila and it is conceivable that, like in mammalian cells, their activation in flies by oxidative stress may induce complex transcriptional responses of both pro-survival and deleterious factors. In this case the integration of these complex responses at the level of the organism will determine the final outcome (protective or deleterious) and, eventually, in the case of a transient stress of limited intensity, the return to an unstressed equilibrium state. Thus the protective or deleterious role of a stress responsive gene cannot be predicted simply but should be uncovered systematically by genetic studies.

Interestingly, in our genetic experiments, halving the dosage of the *Xbp1 *gene resulted in increased sensitivity of flies to paraquat-induced stress. *Xbp1 *is known to be involved in ER stress response in mammals [40]. It has been shown that it is regulated by processing of its mRNA by the C-terminal endonuclease Ire1. Conversely, we observed no transcriptional change of *Xbp1 *in Drosophila challenged with tunicamycin but it is overexpressed in oxidative stress conditions. Our *in vivo *genetic study suggests that this regulation is functionally relevant to oxidative stress protection in Drosophila. Thus *Xbp1 *may protect against different stress conditions through different modes of regulation (transcriptional or post-transcriptional regulation). In agreement to the conservation of this mechanism between flies and mammals, it has been shown recently that, in a mammalian dopaminergic cell line, *Xbp1 *is induced by the parkinsonian mimetic 6-hydroxydopamine which is known to induce oxidative stress [41].

Another gene that affects the flies stress resistance *in vivo *is *Acer*. This gene encodes one of two Drosophila proteins homologous to the mammalian angiotensin converting enzyme (ACE) gene family. Controversial findings have linked *Ace *to stress resistance and aging ([42] and references therein). *Acer *is more similar to the mammalian gene *Ace2*. It has been recently shown that both *Acer *and *Ace2 *are essential regulators of heart function [43]. Interestingly, complete targeted disruption of *Ace2 *in mice results in increased angiotensin II levels and upregulation of hypoxia-induced genes. In Drosophila, the targets of *Acer *are not known and complete loss of function of the gene results in embryonic lethality. We found that halving the dosage of *Acer *in adult flies results in increased sensitivity to paraquat stress. Considering the mammalian data, one hypothesis to explain this result is that heart cells of *Acer*/+ flies may already experience a mild hypoxic stress which sensitizes them to the additional paraquat-induced oxidative stress. Targeted expression of *Acer *in Drosophila heart cells may help to test this hypothesis.

In this genetic study, based on a small subset from the genes found to be regulated by stress in our microarray experiments, we identified genes with no previously known function as *in vivo *modulators of oxidative stress resistance. Since genomic programs steadily increase the number of transposon targeted genes it will become easier to perform this kind of genetic analysis to increase our knowledge of integrated mechanisms of stress resistance in Drosophila.

In conclusion, our data confirm that full genome scanning by microarray experiments and analysis of multiple experimental conditions constitutes a powerful tool to uncover potentially significant biological features that can be subsequently confirmed by genetic experiments.

## Supplementary Material

Additional file 1 For each of the 1368 probe sets identified as stress responsive in our data analysis, we calculated and reported in this table, for each stress condition, the mean ratio <Stress condition> ≤ (AV_stress_*i* / AV_ref_*j*)>*i,j* where AV_stress_*i* and AV_ref_*j* correspond to the average value measured for the *i*th sample in the stress condition and the *j*th sample respectively in the reference condition. To facilitate visual inspection, we used a color code (red corresponding to upregulation, green to downregulation) with thresholds corresponding to fold changes of 1.8 (dark colors), 1.5 (medium) and 1.25 (light). The standard error for each measurement is given in parenthesis. For each probe set, the mean detection *p-value* from MAS5 analysis of reference samples is reported in column 4 and cluster assignment in column 9. . Click here for file

Additional file 2 For stresses induced by a) paraquat (5mM and 15mM experiments), b) H_2_O_2_ or c) tunicamycin we analyzed the distribution in biological processes (as defined by the Gene Ontology (GO) database) of the genes selected by the SAM analysis (responsive genes) and compared it to the same distribution for all the genes significantly detected on our microarrays (analysed genes). We report here the significantly over- or under-represented (P < 0.005) biological process and the number of analysed and responsive genes found inside these classes, for the different stress conditions. The *p-value* P associated to the null hypothesis of no association with a binomial distribution hypothesis is given for each class, (only classes with P < 0.005 were retained). For clarity of the figure some redundant branches of the tree were removed. Color codes for the classes: dark blue: classes present in the 3 stress responses; yellow: classes present in the two oxidative stress responses; green: classes present in paraquat and tunicamycin stress responses; light cyan: classes present in H_2_O_2_ and tunicamycin stress responses. 34 Color code for statistical analysis: orange: underrepresented class, blue: over-represented class. . Click here for file

Additional file 3 For over or under-represented molecular functions classes we report here the number of analyzed (column 3) and responsive genes found inside the 7 groups of clusters A to G (columns 4 to 10, see text for details on the definition of these groups). A schematic response to oxidative and ER stress of the genes included in these groups is given in the first two lines. The number of genes inside each group is given in line 3. A color code identifies cases when the number of genes differs statistically (p<0.005) from a random distribution: orange: under-represented class, blue: over-represented class. Click here for file

Additional file 4 For over or under-represented biological process classes we report here the number of analyzed (column 3) and responsive genes found inside the 7 groups of clusters A to G (columns 4 to 10, see text for details on the definition of these groups). A schematic response to oxidative and ER stress of the genes included in these groups is given in the first two lines. The number of genes inside each group is given in line 3. A color code identifies cases when the number of genes differs statistically (p<0.005)from a random distribution: orange: under-represented class, blue: over-represented class.
Click here for file

Additional file 5 Stress response for the peptidases Click here for file

Additional file 6 List of stress responsive genes detected in aging and hyperoxia, immune or starvation stress experiments were compared with our data. Lines 1 to 3 indicate the number of genes found to be repressed (-), induced (+) or either (total) in the different experiments. Lines 5 to 7 indicate the number of genes in each of these categories found among our 1397 35 stress responsive genes (classes A to F) and the corresponding percentage from the initial number. In lines 8, 9 (respectively 10, 11) the same analysis is reported for genes included in the A (respectively B) classes defined as common stress responsive classes in our analysis. **O_2_, old, infection, starvation**: expression data from the different experiments compared to our data. **All**: List of 26 genes which are responsive to at least 4 stresses in these independent experiments
Click here for file

Additional file 7 Relationships to other stresses Click here for file

Additional file 8 Table 1: Stress response characteristics of clusterized genes.The 1368 probe sets retained after statistical analysis were submitted to a divisive clustering algorithm (SOTA) which predicted 19 clusters. For each probe set *k* inside a cluster we calculated, for each stress condition, the mean ratio R_k_ = <Ln2 (AV_stress_^*i*^ / AV_ref_*^ j ^*)>*i,j* where AV_stress_*^i ^*and AV_ref_*^ j^* denote the average value measured for the *i*^th ^sample in the stress condition and the *j*^th ^sample respectively in the reference condition. The mean of the R_k_ values provides a measurement of the mean intensity of variation for the genes inside a cluster, which is reported in this table. To facilitate visual inspection, we used a color code (red colors corresponding to upregulation, green colors to downregulation) with thresholds corresponding to fold changes of 1.8 (dark colors), 1.5 (medium) and 1.25 (light). The number N of probe sets in each cluster is also reported. From these values we identified groups of clusters (named from A to G) which present close behavior and were used for statistical functional analysis. Clusters corresponding to the common long term stress response (CLTSR) are outlined in red.
Click here for file

Additional file 9 Table 2: Functional analysis of stress responsive genes.For stresses induced by a) paraquat (5mM and 15mM experiments), b) H2O2 or c) tunicamycin we analyzed the distribution in functional classes (as defined by the Gene Ontology (GO) database) of the genes selected by the SAM analysis (responsive genes) and compared it to the same distribution for all the genes significantly detected on our microarrays (analysed genes). We report here the significantly over- or under-represented (P<0.005) molecular functions and the number of analysed and responsive genes found inside these classes, for the different stress conditions. The* p-value* P associated to the null hypothesis of no association with a binomial distribution hypothesis is given for each class, (only classes with P<0.005 were retained). For clarity of the figure some redundant branches of the tree were removed. Color codes for the classes: dark blue: classes present in the 3 stress responses; yellow: classes present in the two oxidative stress responses; green: classes present in paraquat and tunicamycin stress responses; light cyan: classes present in H_2_O_2_ and tunicamycin stress responses. Color code for statistical analysis: orange: under-represented class, blue: over-represented class.
Click here for file

Additional file 10 Table 3: Analysis of stress responses for members of some functional classes.From the 1368 stress responsive probe sets we extracted the subsets associated with genes annotated in the GO database as a) glutathione transferases (GO:0004364), b) P450 cytochromes (from list at http://p450.antibes.inra.fr/) and c) proteasome component (GO:0004299). For each probe set k within one of these subsets, we calculated and reported in this table, for each stress condition, the mean ratio <Stress condition >k =<(AVstressi / AVref j)>i,j where AVstressi and AVrefj correspond to the average value measured for the ith sample in the stress condition and the jth sample respectively in the reference condition. To facilitate visual inspection, we used a color code (red corresponding to upregulation, green to downregulation) with thresholds corresponding to fold changes of 1.8 (dark colors), 1.5 (medium) and 1.25 (light). The standard error for each measurement is given in parenthesis. For each probe set, the mean detection p-value from MAS5 analysis of reference samples is reported in column 3 and cluster assignment in column 8. In column 9 additional information is reported for each class: in a) we indicate the GT class deduced from sequence comparison with human and mouse GTs and from [44] (D: delta, O: omega, T: theta, T2: distantly related to theta, Z: zeta); in b) the name of the genes are reported; in c) we indicate the proteasome subunit to which the genes defined in column 2 belong. Note that in c) a large number of genes not retained by SAM analysis (without cluster number) seem to be upregulated in P15 condition. Genes used for comparison between microarray and quantitative RT-PCR (Fig. 3) are outlined in bold character.
Click here for file

Additional file 11 Table 4: Analysis of mutant flies' resistance to paraquat-induced oxidative stress. Paraquat resistance of 29 mutant lines was assayed in three independent experiments as described in Fig. 4. The survival data were submitted to a log-rank statistical analysis by comparison with *w*/+ reference flies. The results are presented in this table. Column 1 contains the tested genotype (same conventions as in Fig. 4a : Bloomington line numbers). The corresponding genotypes are described in column 6. The symbol for the gene affected is reported in column 2. Information from FlyBase about the allele used in this study is given in column 7 with a one character code: A: amorph; H: hypomorph; N: non complementation of deficiency; L: letal; R: recessive mutation; 5: insertion in the 5' regulatory region, 5'UTR or intron; C: insertion in the coding region. Column 8 indicates whether the tested line was outcrossed or not before the test. Column 4 is the result of a log-rank analysis of the second survival experiments shown in figure 4. A confidence index which refers to the mean of log10 (p-log-rank) for the three experiments is given in column 5. We considered that a strain had a significant effect on survival under oxidative stress conditions when this confidence index was lower than -3 and at least two experiments presented p-log-rank < 0.001. Under these stringent conditions 13 genotypes are shown to confer resistance (R) or sensitivity (S) to paraquat as indicated in column 3. Click here for file
