# Supplementary material for: Genome wide analysis of common and specific stress responses in adult drosophila melanogaster
Source: BMC Genomics. 2004 Sep 30;5:74. doi: 10.1186/1471-2164-5-74 (PMC526417; doi:10.1186/1471-2164-5-74)
Supplement: Additional file 7 — Relationships to other stresses [file 1471-2164-5-74-S7.doc]

# Relationships to other stresses

Several transcriptome analysis on Affymetrix chips in response to various stresses (immune stress [1], starvation [2] and, during the submission of this work, hyperoxia and aging [3] have been recently published. A full comparison of these data with ours would require a full treatment of the raw data for common normalization, similar statistical analysis and complete clusterization and could be criticized as mixing data from heterogeneous conditions (strains, age, rearing conditions, ...).

Nevertheless, simpler analysis may be performed by using the lists of stress-responsive genes provided from the above works and checking their transcriptional profile in our experiments. We can then determine the percentage *P* of genes from these lists which are included in the classes A to F defined above from our clustering analysis. Several conclusions can be drawn from such a crude analysis (Table S5): 1) as expected a high correlation (*P*=69%) was observed between hyperoxia and paraquat stress response 2) 31% of age-responsive genes are detected in our experiments which further confirms the correlation between aging and oxidative stress already reported by other teams [3, 4]. 3) while a large fraction (*P*=43%) of immune stress-responsive genes are also affected in paraquat stress, the two stresses have opposite effects for some 33% fraction of these genes. We were unable to uncover any obvious functional differences between these different classes. 4) the weaker correlations observed with the starvation data may result from the induction of the starvation stress at a larval stage. Starvation experiments performed on adult flies are clearly needed to confirm these differences.

From this simple analysis, 26 genes are responsive to at least 4 stresses in these independent experiments (Tab. S5) and could represent the core of a very general stress response. Among these genes the Thor translational repressor may play an important role to reduce translational in case of cellular stress as observed in yeast [5].

1. E De Gregorio, PT Spellman, GM Rubin, B Lemaitre: **Genome-wide analysis of the Drosophila immune response by using oligonucleotide microarrays**. *Proc Natl Acad Sci U S A* 2001, **98**:12590-5.

2. I Zinke, CS Schutz, JD Katzenberger, M Bauer, MJ Pankratz: **Nutrient control of gene expression in Drosophila: microarray analysis of starvation and sugar-dependent response**. *Embo J* 2002, **21**:6162-73.

3. GN Landis, D Abdueva, D Skvortsov, J Yang, BE Rabin, J Carrick, S Tavare, J Tower: **Similar gene expression patterns characterize aging and oxidative stress in Drosophila melanogaster**. *Proc Natl Acad Sci U S A* 2004, **101**:7663-8.

4. S Zou, S Meadows, L Sharp, LY Jan, YN Jan: **Genome-wide study of aging and oxidative stress response in Drosophila melanogaster**. *Proc Natl Acad Sci U S A* 2000, **97**:13726-31.

5. HC Causton, B Ren, SS Koh, CT Harbison, E Kanin, EG Jennings, TI Lee, HL True, ES Lander, RA Young: **Remodeling of yeast genome expression in response to environmental changes**. *Mol Biol Cell* 2001, **12**:323-37.
